# A New Hydrogen Sensor Based on SNS Fiber Interferometer with Pd/WO_3_ Coating

**DOI:** 10.3390/s17092144

**Published:** 2017-09-18

**Authors:** Jin-xin Shao, Wen-ge Xie, Xi Song, Ya-nan Zhang

**Affiliations:** 1 College of Information Science and Engineering, Northeastern University, Shenyang 110819, China; shaojinxin@stumail.neu.edu.cn (J.S.); xiewenge@stumail.neu.edu.cn (W.X.); songxi@stumail.neu.edu.cn (X.S.); 2 State Key Laboratory of Synthetical Automation for Process Industries, Shenyang 110819, China

**Keywords:** fiber interferometer, no core fiber (NCF), hydrogen sensor, Pd/WO_3_ film

## Abstract

This paper presents a new hydrogen sensor based on a single mode–no core–single mode (SNS) fiber interferometer structure. The surface of the no core fiber (NCF) was coated by Pd/WO_3_ film to detect the variation of hydrogen concentration. If the hydrogen concentration changes, the refractive index of the Pd/WO_3_ film as well as the boundary condition for light propagating in the NCF will all be changed, which will then cause a shift into the resonant wavelength of interferometer. Therefore, the hydrogen concentration can be deduced by measuring the shift of the resonant wavelength. Experimental results demonstrated that this proposed sensor had a high detection sensitivity of 1.26857 nm/%, with good linearity and high accuracy (maximum 0.0055% hydrogen volume error). Besides, it also possessed the advantages of simple structure, low cost, good stability, and repeatability.

## 1. Introduction

Nowadays, energy has become an increasingly important issue in human development. Scientists believe that hydrogen will become another new source of energy to replace petroleum [1,2]. Although hydrogen has a lot of advantages, such as reproducibility, no pollution, high burning ratio, and so on [3], it is explosive and flammable due to its high diffusion coefficient (0.16 cm^2^/s in air), low ignition energy (0.018 mJ), and high combustion heat (285.8 kJ/mol). For example, when hydrogen leaks into the air and the concentration of it reaches 4~74.5%, the mixture will be in an explosive state at room temperature and atmospheric pressure, and it will also be detonated when exposing to open flame [4]. Thus the wide application of hydrogen energy is limited. Therefore, it is of great value to develop a safe and reliable hydrogen sensor with high sensitivity to monitor the hydrogen concentration in real time.

Hydrogen sensors can be divided into catalyst (Pellistor) type, thermal conductivity type, electrochemical type, optical fiber type, and so on [5]. Pellistor-typed catalytic hydrogen sensors are based on the theory that combustion of hydrogen and oxygen on the sensor surface can release heat for measuring. However, they have a high error rate, high power consumption, and a high risk of explosion [6]. Thermal conductivity typed sensors for hydrogen analysis were initially used for airships since 1913. The principle of this sensor is to measure heat loss from the detector to the surrounding gas [7]. The detection range can often cover 1–100%, but cannot be used when the concentration of hydrogen is low. Electrochemical hydrogen sensors use the solid electrolyte to react with hydrogen and then to change the current in the circuit [8]. Finally, the concentration of hydrogen can be determined by observing the change of the output voltage. Using an electrical signal, however, is easy to cause an explosion and compromise the measurement. By contrast, the measurement principle of an optical fiber hydrogen sensor is that the reaction between hydrogen and hydrogen sensitive material deposited on the fiber will change some properties of the hydrogen sensitive material, and then the wavelength or intensity of optical signal in the optical fiber will also be changed [9]. By observing the variations of the optical signal, the hydrogen concentration can be deduced. It should be mentioned that the most likely reason for high hydrogen leak explosions is due to some form of electrostatic charging [10]. However, due to the intrinsic safety of light signals, this problem can be avoided in optical fiber hydrogen sensors even when the optical fiber is broken. So it is obvious that optical fiber hydrogen sensors can realize the measurement of hydrogen concentration with high safety and accuracy.

Broadly speaking, the measurement technologies of optical fiber hydrogen sensors contain a micro-mirror, evanescent field, surface plasmon resonance (SPR), interference, fiber Bragg grating (FBG), and so on [11]. A micro-mirror sensor was first put forward by M. A. Butler in 1994 [12]. The palladium film coated on the end of optical fiber would react with hydrogen and then the reflectivity of the film will be changed. So the relationship between hydrogen concentration and the intensity of reflected light can be established. The evanescent field sensor was first proposed by Tabib Azar who coated the sensitive material on the fiber to cause an intensity change in the optical signal [13]. However, the light intensity will easily be interrupted by surroundings, which will then induce a measurement error. The surface plasmon resonance (SPR) hydrogen sensor is realized by monitoring the shift of the resonance spectrum under the condition that the hydrogen sensitive film is properly coated onto the surface of a metal film [14]. This sensor has a high sensitivity, but the deposition of the essential metal film is expensive and difficult. The interference sensor was first proposed by Butler who used an optical fiber Mach–Zehnder (MZ) interferometer sensor [15]. When the Pd layer, which is deposited on the sensor, reacts with hydrogen, its volume will increase and the refractive index will decrease, which can cause a phase change in the optical signal [16]. For the FBG coated with the sensitive materials (for example Pd), the reflecting signal of FBG will be changed when the Pd film reacts with hydrogen. The hydrogen sensor based on FBG has a fast response speed, but the measurement sensitivity is relatively low. By utilizing the polishing, etching, or tapering technology, the measurement sensitivity can be enhanced, but the life of sensor will be shortened [17]. In comparison to other technologies, the sensor based on interference theory possesses particular advantages of simple structure, low cost, high sensitivity, and so on. In recent years, a lot of interferometer structures have been proposed [18]. Upon these methods, this paper provides a new thought of hydrogen sensor by simply splicing a no core fiber between two single mode fibers. The surface of the NCF is coated with Pd/WO_3_ film to detect the variation of hydrogen concentration. Simulation and experimental results demonstrated that this simple structure can realize a high sensitivity of 1.26857 n m/%, with good linearity, high stability, and good responsiveness.

## 2. Structure and Principle of Sensor

**Figure 1:**
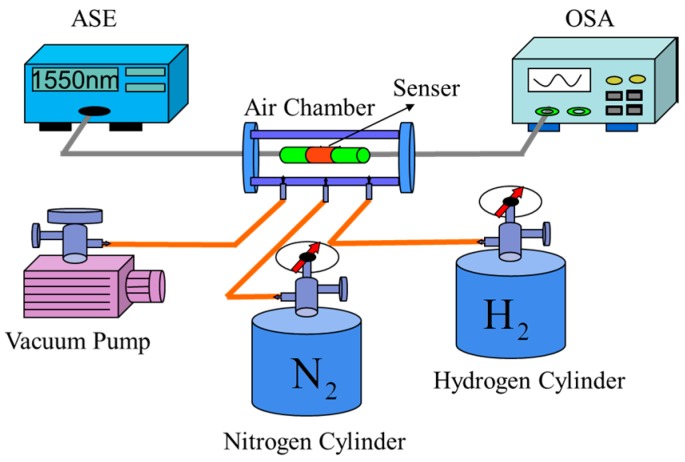
Schematic structure of hydrogen sensing system.

**Figure 2:**
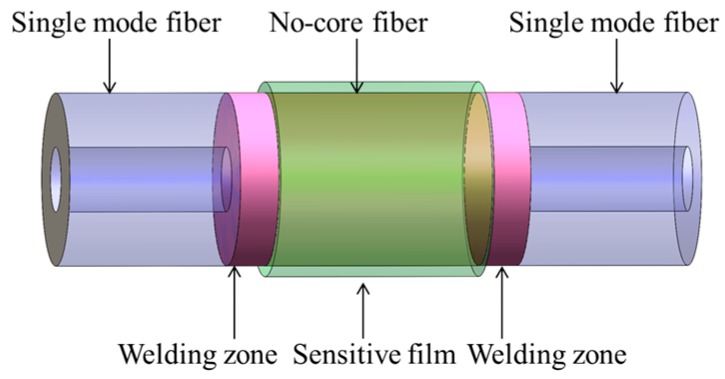
Specific structure of SNS sensing structure.

Figure 1 shows the schematic structure of our sensing system. The structure consists of an amplified spontaneous emission source (ASE) with an operating wavelength ranging from 1520 nm to 1570 nm, an optical spectrum analyzer (OSA) with spectral resolution of 0.02 nm, and a hydrogen sensing probe. The hydrogen sensing head is constituted by two sections of single mode fiber (SMF) and one section of no core fiber (NCF) with Pd/WO_3_ coating, which is shown in Figure 2. The core and cladding diameters of SMF and NCF that used are 9/125 μm and 125/125 μm, respectively. These three fibers were spliced together by using a commercial fusion machine. In this structure, the light emitted from ASE feeds into the SMF, and propagates with the fundamental mode. When it travels from SMF to the NCF, multiple high-order modes are excited. Then the fundamental and high-order modes meet in the second welding zone and interfere with each other [19]. Finally, the interference spectrum is received by the OSA. To measure the hydrogen sensing property of the proposed sensor in an experiment, the sensing head was inserted into a closed air chamber. The hydrogen concentration of the air chamber can be controlled by adjusting the flow velocities of hydrogen and nitrogen with two mass flow controllers. In the measurement, the air chamber is evacuated to a vacuum state by a vacuum pump each time before the hydrogen concentration is changed. By this way, we can control the hydrogen concentration in the air chamber accurately.

According to multiple high-order LP (linear polarization) modes propagation theory, *β_m_* and *β_n_* are assumed to be the longitudinal propagation constants for *m* and *n* order modes in the NCF. So their relationship can be expressed as [20]

(1) $$\beta_{m}-\beta_{n}=\frac{u_{m}^{2}-u_{n}^{2}}{2k_{0}a^{2}n_{core}}$$

where *a* is the core radius of the NCF, *n_core_* is the core refractive index of NCF, *k*_0_ = 2π/*λ* is the is free-space wave number, *u_m_* and *u_n_* are normalized transverse propagation constants and they can be calculated by the next formula [21]

(2) $$u_{x}=(2x-0.5)\cdot \pi /2$$

If the phase difference between the two modes is an integral multiple of π, then the constructive interference of these modes occurs. Particularly, the resonant dips of the interference spectrum are generated when the phase difference is an odd multiple of π [22], namely

(3) $$\left(\beta_{m}-\beta_{n}\right)\cdot L=(2N+1)\;\pi$$

By solving the combined equations of Formulas (1) and (3), the wavelengths of resonant dips can be expressed as

(4) $$\lambda =\frac{8(2N+1)n_{core}a^{2}}{(m-n)[2(m+n)-1]L}$$

where *L* is the length of NCF, and N is a natural number.

So for different constructive interferences, it is easy to get the wavelength shift of resonant spectrum

(5) $$\Delta \lambda =\lambda_{N}-\lambda_{N-1}=\frac{16n_{core}a^{2}}{(m-n)\cdot [2(m+n)-1]\cdot L}$$

Therefore, by coating Pd/WO_3_ film on the surface of the NCF, we can establish a relationship between hydrogen concentration and the interference spectrum. A common method called the sol-gel method is widely used to manufacture Pd/WO_3_ film, and the film can be coated on the surface of NCF by dip-coating method. Both of these methods established in our group. In our previous work, we illustrated these methods in detail [23]. These methods have the advantages of low cost, simple fabrication, and high success ratio. Both palladium and WO_3_ have good hydrogen sensitivity: palladium can absorb hydrogen with high selectivity, while WO_3_ film has good adhesion and mechanical properties [24]. So if the palladium and WO_3_ film exposed to the hydrogen, with palladium as the catalyst, hydrogen will be absorbed and decomposed. Hydrogen atoms which transferred to the surface of WO_3_ by palladium will spread along the inner holes of WO_3_, and then cause the variation of both volume and refractive index of Pd/WO_3_ film. By coating Pd/WO_3_ on the surface of NCF, the refractive index and stain of NCF surface will change with the variation of hydrogen concentration. However, the variations of *n_core_*, *L*, and *a* of NCF will change little and can be ignored in the theory. However, the propagation constants and mode field will be reformed when the refractive index of the Pd/WO_3_ film is changed. From Formula (3), when the propagation constants are changed, the phase condition of the constructive (or destructive) interference will alter and the natural number, *N*, will also change. Therefore, the main reason caused the wavelength difference is the variation of the natural number *N*.

In summary, if the hydrogen concentration changes, the refractive index of the sensitive film will also be changed. Then the boundary condition for light propagating in the NCF will also change and cause the shift in resonant wavelength. It has also been proven that the resonant wavelength of this SNS interferometer structure has an approximately linear change with the variation of refractive indices of the sensitive film [25]. So if the refractive index of the sensitive film is linearly proportional to the hydrogen concentration, this sensor will gain a good linearity, and it will be proved by the experiment in the following.

## 3. Simulation Analysis

**Figure 3:**
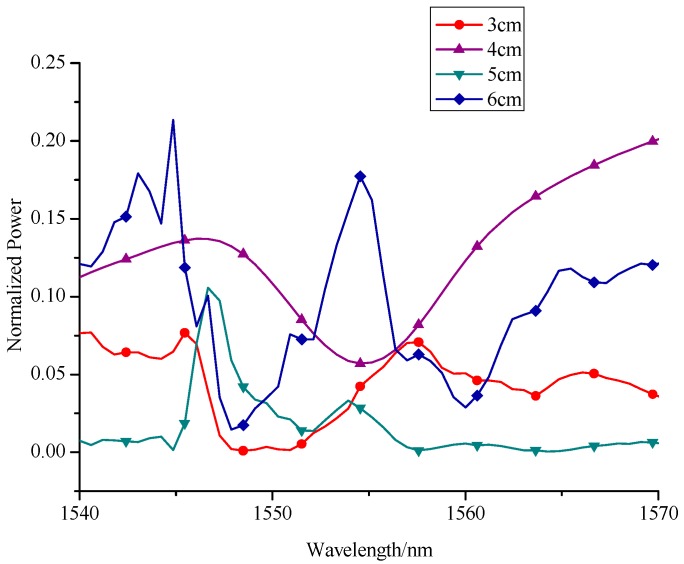
Simulation spectra of SNS interferometer with different lengths of NCF.

From the above analyses, it is obvious that this interferometer structure must create an interference phenomenon to obtain the hydrogen concentration. Besides, the larger the wavelength shift in resonance spectrum is, the higher the hydrogen sensor sensitivity will be. So we should first ensure the length of NCF to obtain a better interferometer effect and a higher sensitivity. In scientific research, modeling and simulation are also important parts to prove this hypothesis. So we used professional simulation software called Rsoft to imitate how the resonant spectrum will be influenced when the length of NCF changes. As shown in Figure 3, when the length of NCF changes from 3 cm to 6 cm with an interval of 1 cm, the simulated interference spectra have different forms, which is mainly due to the final interference spectrum being a combination of the interference spectra of different high-order modes and fundamental mode. Therefore, most interference spectra contain many resonant dips of different shapes. For different lengths of NCF, the high-order modes that generate interference may be different, so the interference spectra are also different. Particularly, the interference spectrum with NCF length of 4 cm has only one resonant dip in the wavelength range of 1540–1570 nm, which is mainly due to there being only one high-order mode to interfere with the fundamental mode in this situation. This smooth and pure spectrum is beneficial for sensors to observe the wavelength shift along with hydrogen concentration variation. Therefore, we will further investigate the refractive index property of this interferometer with NCF length around 4 cm in the following sections.

**Figure 4:**
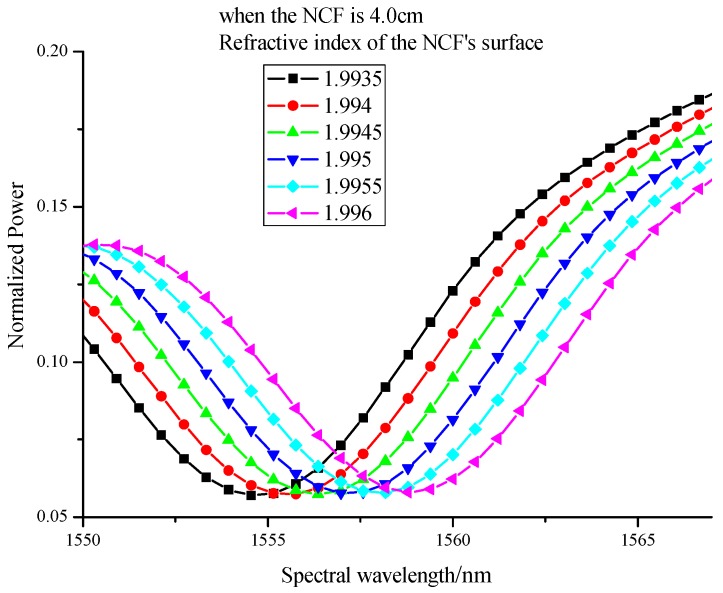
Resonant spectra of interferometer under different refractive indices when the length of NCF is 4 cm.

**Figure 5:**
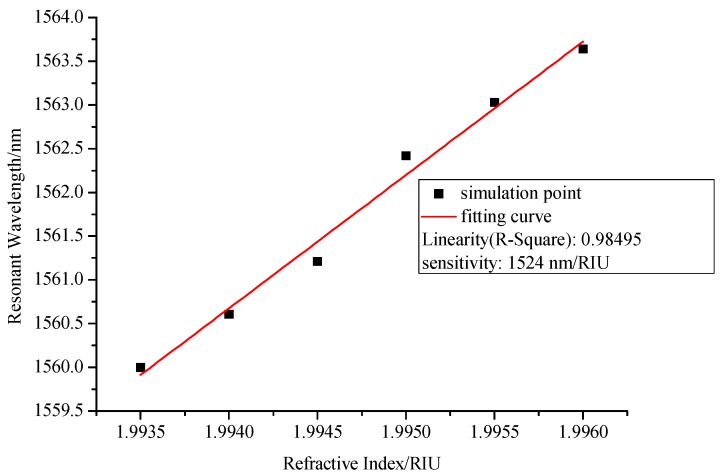
Relationship between resonant wavelength and refractive indices when the length of NCF is 4 cm.

As shown in Figure 4, the resonant wavelength of interferometer will shift to the long wavelength direction when the refractive index of the layer increases, which is consistent with the theoretical analysis. The relationship between refractive indices and resonant wavelength can be seen in Figure 5, which shows high sensitivity of 1524 nm/RIU and good linearity of 0.98495.

**Figure 6:**
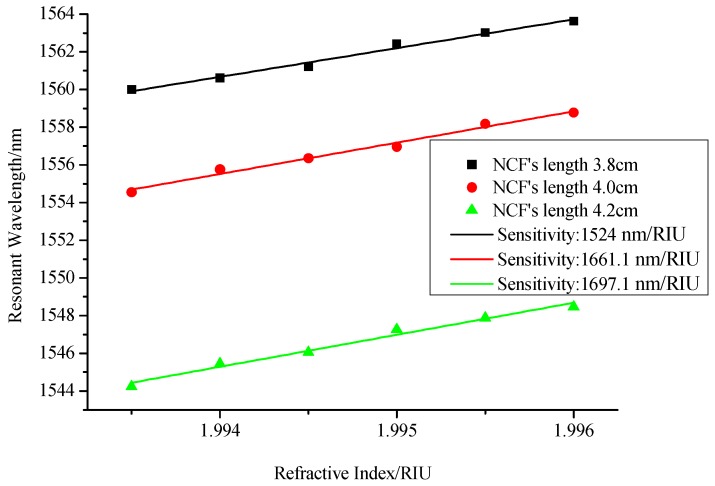
Relationship between resonant wavelength and refractive indices with different lengths of NCF.

Then, considering that the length of NCF is difficult to control precisely, we compared the refractive index sensitivity of interferometer when the length of NCF is slightly changed from 3.8 cm to 4.0 cm and then to 4.2 cm. As shown in Figure 6, with the increase of the length of NCF, the change of refractive index sensitivity is very little. So considering the convenience of sensor fabrication and the error of the welding zone, it is reasonable to choose 4.0 cm as the length of NCF in the following experimental study.

## 4. Experimental Test

**Figure 7:**
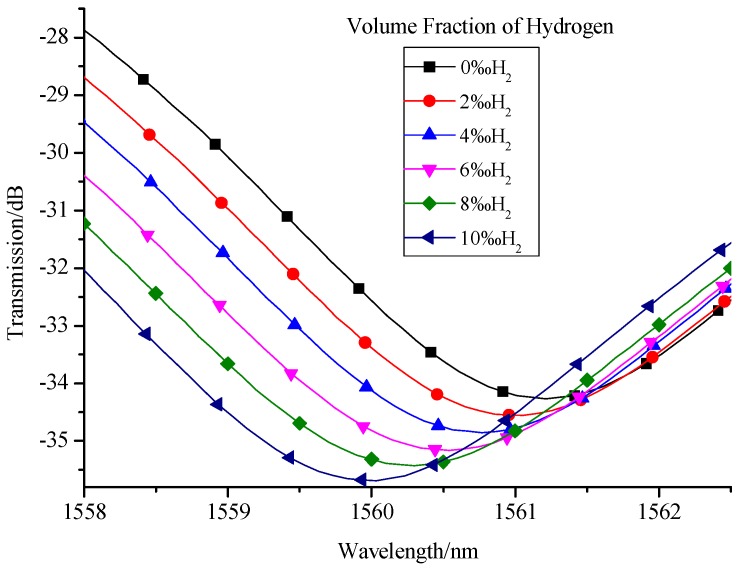
Experimental spectra of sensor for different hydrogen concentrations.

**Figure 8:**
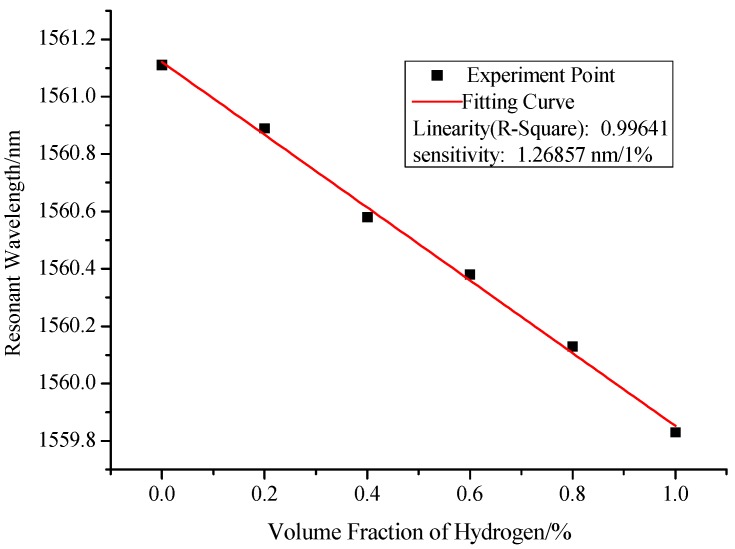
Relationship between resonant wavelength and hydrogen concentration.

After implementing the procedures we designed, we can get the interference spectra of the sensor for different hydrogen concentrations in experiment, as shown in Figure 7. With the increase in hydrogen concentration, the resonant wavelength shifts in short wavelength direction. This is because that the refractive index of Pd/WO_3_ film will decrease when the hydrogen concentration is increasing. Therefore, the experimental result is consistent with the simulation result as shown in Figure 4. Similarly, by quantifying the relationship between the resonant wavelength and hydrogen concentration, as shown in Figure 8, we can get the measurement sensitivity of hydrogen concentration (1.26857 nm/%) with good linearity of 0.99641. The response time of this kind of sensor primarily depends on the sensitivity of the material that is used for sensing. The Pd/WO_3_ based hydrogen sensor will enter into the steady state in no more than 30 min, this has been proven in our previous manuscript [26]. Besides, the response time of hydrogen sensor based on Pd/WO_3_ may be improved by UV-light irradiation [27] or doping graphene quantum dots [28], which will be tested in our future work.

**Figure 9:**
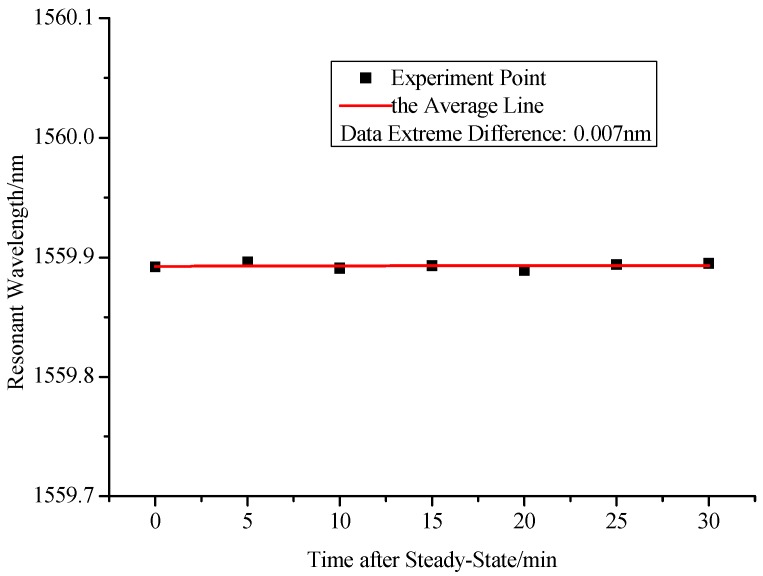
Stability curve of the proposed hydrogen sensor.

To investigate the stability of the proposed hydrogen sensor, we observed the resonant wavelength of sensor every 5 min when the hydrogen concentration of air chamber is stable at 1% for 30 min. From Figure 9, we can see that the maximum change of the resonant wavelength is 0.007 nm, which will cause only 0.0055% hydrogen volume error. Therefore, we can say that the proposed sensor has good stability. Besides, it has been demonstrated that humidity has little influence on this kind of sensor [29], which means that the humidity compensation may not be required for most of hydrogen sensing applications. Besides, a Teflon layer can be used to decrease the effect of H_2_O on the hydrogen sensitive coating layer if the humidity variation is large [30].

**Figure 10:**
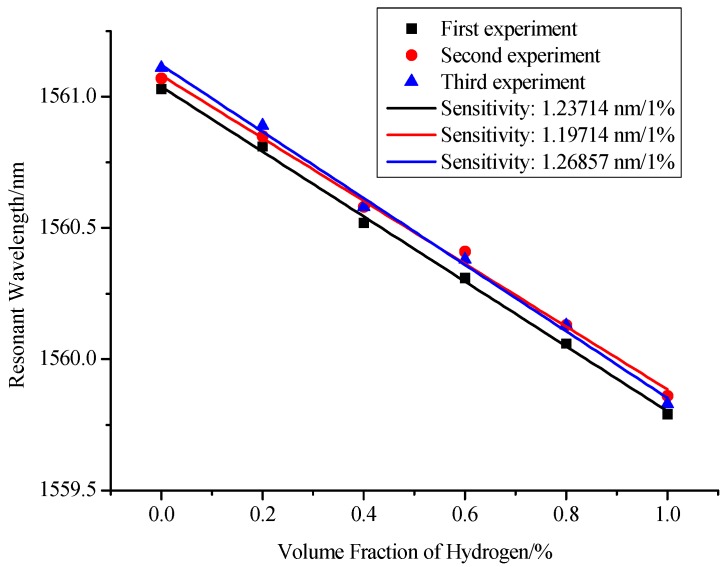
Repeatability curve of the proposed hydrogen sensor.

Then we further investigate the repeatability of the sensor. For one sensor, we conducted the same experimental tests three times. Then we got the sensitivity curves of sensor for each test, as shown in Figure 10. From this figure, we can confirm that the proposed sensor also has a good repeatability. We can also see that the three lines are almost parallel but the intercepts of these lines increase regularly. Namely, the origins of the resonant wavelengths are not the same in each measurement, which is mainly due to that the temperature is different in each test. This problem can be resolved by analyzing the relationship between the change of the resonant wavelength and the change of hydrogen concentration. Besides, this temperature influence can also be compensated by measuring the hydrogen concentration and temperature, simultaneously [31].

## 5. Conclusions

In summary, this paper proposed an in-line SNF interferometer structure to measure hydrogen concentration. Then the hydrogen sensing properties of this structure had been proven by theoretical derivation, simulation calculation, and experimental test. Results demonstrated that the proposed sensor could achieve high-sensitivity hydrogen concentration detection (1.26857 nm/%). Also, the proposed sensor shows high precision and accuracy (maximum 0.0055% hydrogen volume error). Meanwhile, it has the advantages of simple structure, low cost, good linearity, stability, and repeatability.

## References

[1] Jacobson M.Z., Colella W.G., Golden D.M. (2005). Clearing the air and improving health with hydrogen fuel-cell vehicles. Science.

[2] Dedes E.K., Hudson D.A., Turnock S.R. (2012). Assessing the potential of hybrid energy technology to reduce exhaust emissions from global shipping. Energy Policy.

[3] Dincer I. (2000). Renewable energy and sustainable development: A crucial review. Renew. Sustain. Energy Rev..

[4] Silva S.F., Coelho L., Frazão O. (2012). A review of palladium-based fiber-optic sensors for molecular hydrogen detection. IEEE Sens. J..

[5] Hübert T., Boon-Brett L., Black G., Banach U. (2011). Hydrogen sensors—A review. Sens. Actuators B Chem..

[6] Firth J.G., Jones A., Jones T.A. (1973). The principles of the detection of flammable atmospheres by catalytic devices. Combust. Flame.

[7] Pollak-Diener G., Obermeier E. (1993). Heat-conduction microsensor based on silicon technology for the analysis of two-and three-component gas mixtures. Sens. Actuators B Chem..

[8] Korotcenkov G., Han S.D., Stetter J.R. (2009). Review of electrochemical hydrogen sensors. Chem. Rev..

[9] Dai J., Zhu L., Wang G. (2017). Optical fiber grating hydrogen sensors: A review. Sensors.

[10] Astbury G.R., Hawksworth S.J. (2007). Spontaneous ignition of hydrogen leaks: A review of postulated mechanisms. Int. J. Hydrogen Energy.

[11] Yang M., Dai J. (2014). Fiber optic hydrogen sensors: A review. Photonic Sens..

[12] Butler M.A. (1994). Micromirror optical-fiber hydrogen sensor. Sens. Actuators B Chem..

[13] Tabib A.M., Sutapun B., Petrick R. (1999). Highly sensitive hydrogen sensors using palladium coated fiber optics with exposed cores and evanescent field interactions. Sens. Actuators B Chem..

[14] Bevenot X., Trouillet A., Veillas C. (2001). Surface plasmon resonance hydrogen sensor using an optical fibre. Meas. Sci. Technol..

[15] Butler M.A., Ginley D.S. (1988). Hydrogen sensing with palladium-coated optical fibers. J. Appl. Phys..

[16] Yang M., Sun Y., Zhang D. (2010). Using Pd/WO_3_ composite thin films as sensing materials for optical fiber hydrogen sensors. Sens. Actuators B Chem..

[17] Dai J., Yang M., Yu X. (2012). Greatly etched fiber Bragg grating hydrogen sensor with Pd/Ni composite film as sensing material. Sens. Actuators B Chem..

[18] Cai L., Zhao Y., Li X.G. (2015). Applications of modal interferences in optical fiber sensors based on mismatch methods. Instrum. Sci. Technol..

[19] Raghunandhan R., Chen L.H., Long H.Y. (2016). Chitosan/PAA based fiber-optic interferometric sensor for heavy metal ions detection. Sens. Actuators B Chem..

[20] Mohammed W.S., Mehta A., Johnson E.G. (2004). Wavelength tunable fiber lens based on multimode interference. J. Lightwave Technol..

[21] Zhao Y., Cai L., Li X.G., Meng F.C., Zhao Z. (2014). Investigation of the high sensitivity RI sensor based on SMS fiber structure. Sens. Actuators A Phys..

[22] Zhao Y., Li X.G., Cai L. (2016). A reflective intensity modulated fiber tilt angle sensor based on an all-photonic crystal fiber interferometer. Sens. Actuators A Phys..

[23] Zhang Y.N., Peng H., Zhou T., Zhang L., Zhang Y., Zhao Y. (2017). Hydrogen sensor based on high-birefringence fiber loop mirror with sol-gel Pd/WO_3_ coating. Sens. Actuators B Chem..

[24] Fardindoost S., Rahimi F., Ghasempour R. (2010). Pd doped WO_3_ films prepared by sol-gel process for hydrogen sensing. Int. J. Hydrogen Energy.

[25] Liu X., Zhang X., Liu Y. (2016). Multi-point fiber-optic refractive index sensor by using coreless fibers. Opt. Commun..

[26] Zhang Y., Wu Q., Peng H. (2016). Photonic crystal fiber modal interferometer with Pd/WO_3_ coating for real-time monitoring of dissolved hydrogen concentration in transformer oil. Rev. Sci. Instrum..

[27] Zhong X.X., Yang M.H., Huang C.J., Wang G.P., Dai J.X., Bai W. (2016). Water photolysis effect on the long-term stability of fiber optic hydrogen sensor with Pt/WO_3_. Sci. Rep..

[28] Fardindoost S., Zad A.I., Hosseini Z.S., Hatamie S. (2016). Detecting hydrogen using graphene quantum dots/WO_3_ thin films. Mater. Res. Express.

[29] Dai J., Yang M., Yang Z., Li Z., Wang Y., Wang G., Zhang Y., Zhuang Z. (2014). Performance of fiber Bragg grating hydrogen sensor coated with Pt-loaded WO_3_ coating. Sens. Actuators B Chem..

[30] Coelho L., de Almeida J., Santos J.L. (2015). Fiber optic hydrogen sensor based on an etched Bragg grating coated with palladium. Appl. Opt..

[31] Wang M., Wang D.N., Yang M., Cheng J., Li J. (2014). In-line Mach-Zehnder Interferometer and FBG with Pd film for simultaneous hydrogen and temperature detection. Sens. Actuators B Chem..

